# *cis*,*cis*,*cis*-Di­chlorido­bis­(*N* ^4^,*N* ^4^-di­methyl­pyridin-4-amine-κ*N* ^1^)bis­(dimethyl sulfoxide-κ*S*)ruthenium(II)

**DOI:** 10.1107/S2414314624001913

**Published:** 2024-03-06

**Authors:** Esther H. Park, Sarah M. Ortiz, Todd K. Liang, Bradley W. Smucker

**Affiliations:** a Austin College, 900 N Grand, Sherman, TX 75090, USA; Benemérita Universidad Autónoma de Puebla, México

**Keywords:** crystal structure, coordination compound, ruthenium(II), DMAP, dmso

## Abstract

The structure of the title compound, [RuCl_2_(C_7_H_10_N_2_)_2_(C_2_H_6_OS)_2_], has monoclinic (*P*2_1_/*n*) symmetry. The Ru—N distances of the coordination compound are influenced by the *trans* chloride or di­methyl­sulfoxide-κ*S* ligands. The mol­ecular structure exhibits disorder for two of the terminal methyl groups of a dimethyl sulfoxide ligand.

## Abstract 2

In the structure of the title compound, the Ru—N distances of the DMAP ligands are influenced by the *trans* chloride or di­methyl­sulfoxide-κ*S* ligands.

## Graphical abstract

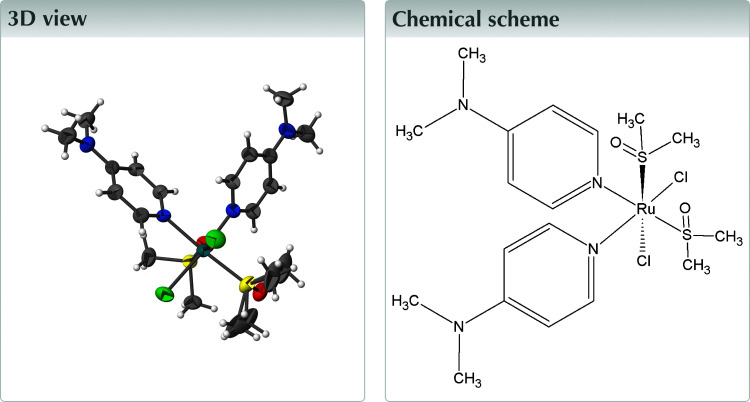

## Structure description

**Figure 1:**
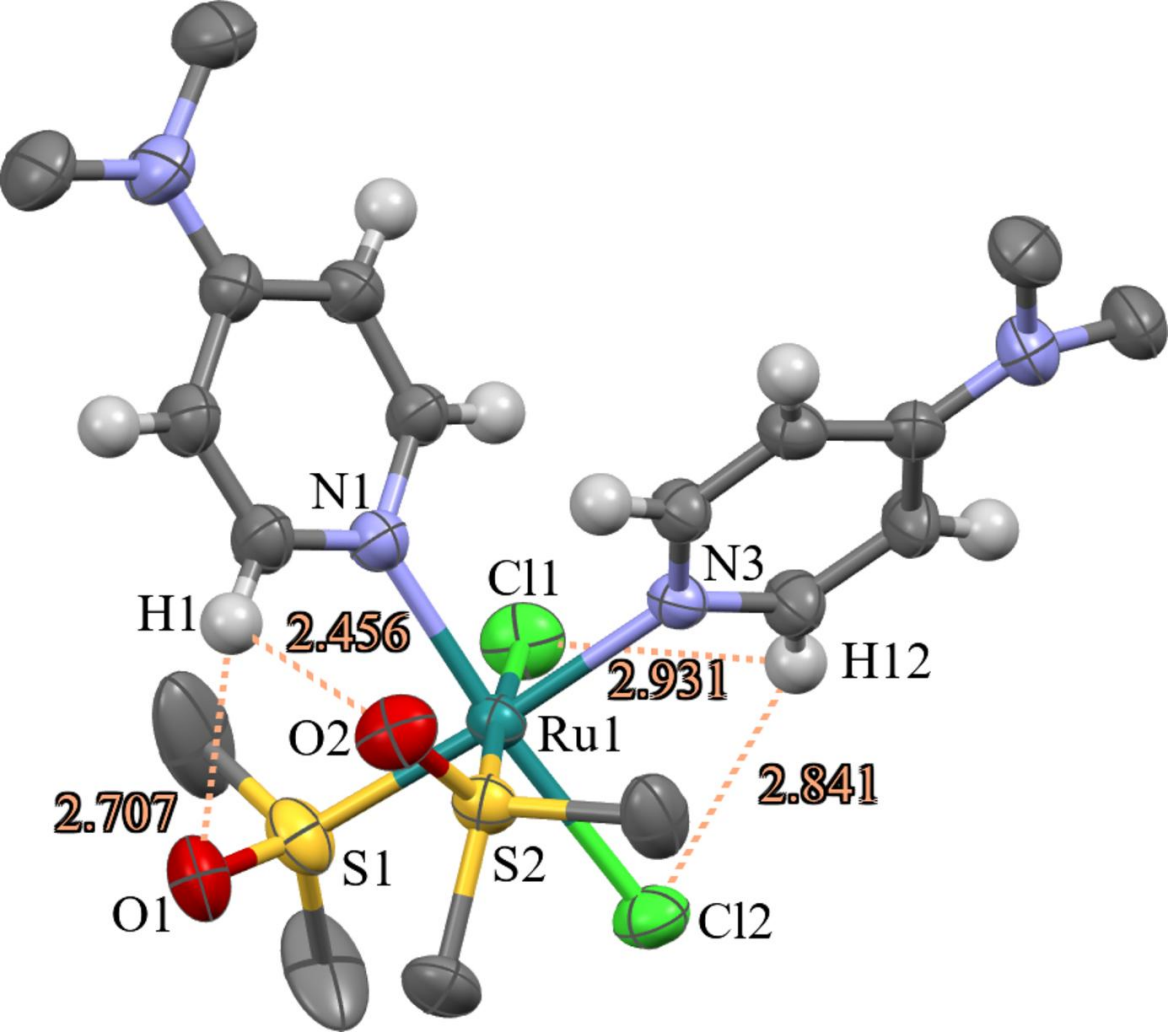
Displacement ellipsoid (50% probability level) representation of the title complex with intra­molecular H⋯O and H⋯Cl distances given (disorder and methyl hydrogen atoms omitted for clarity).

Both symmetry-related Δ and Λ enanti­omers are present in the unit cell. The ruthenium(II) complex has the oxygen atoms of the dimethyl sulfoxide (dmso) ligands positioned in the same general direction toward an H1 atom of a DMAP (*N*,*N*-di­methyl­pyridin-4-amine) ligand with intra­molecular distances of 2.456 (H1⋯O2) and 2.707 Å (H1⋯O1) (Fig. 1 ▸). The two DMAP ligands are both tilted to position the α-hydrogen atoms of the pyridyl rings to inter­act with the aforementioned oxygen atoms of the dmso ligand or the chlorido ligands with distances of 2.841 (H12⋯Cl2) and 2.931 Å (H12⋯Cl1) (Fig. 1 ▸). A comparison between the Ru—N distances for the coordinating DMAP ligands reveals a greater *trans*-influence by the S atom from the dmso ligand [Ru—N3 = 2.150 (4) Å] than by the chloride ligand [Ru—N1 = 2.117 (5) Å]. This influence by the S and Cl atoms agrees with the Ru—N distances found in the crystal structure of a similar Ru^II^ complex containing pyridine instead of DMAP, namely *cis,cis,cis*-[RuCl_2_(dmso-κ*S*)_2_(py)_2_] (Trivedi *et al.*, 2010 ▸).

**Figure 2:**
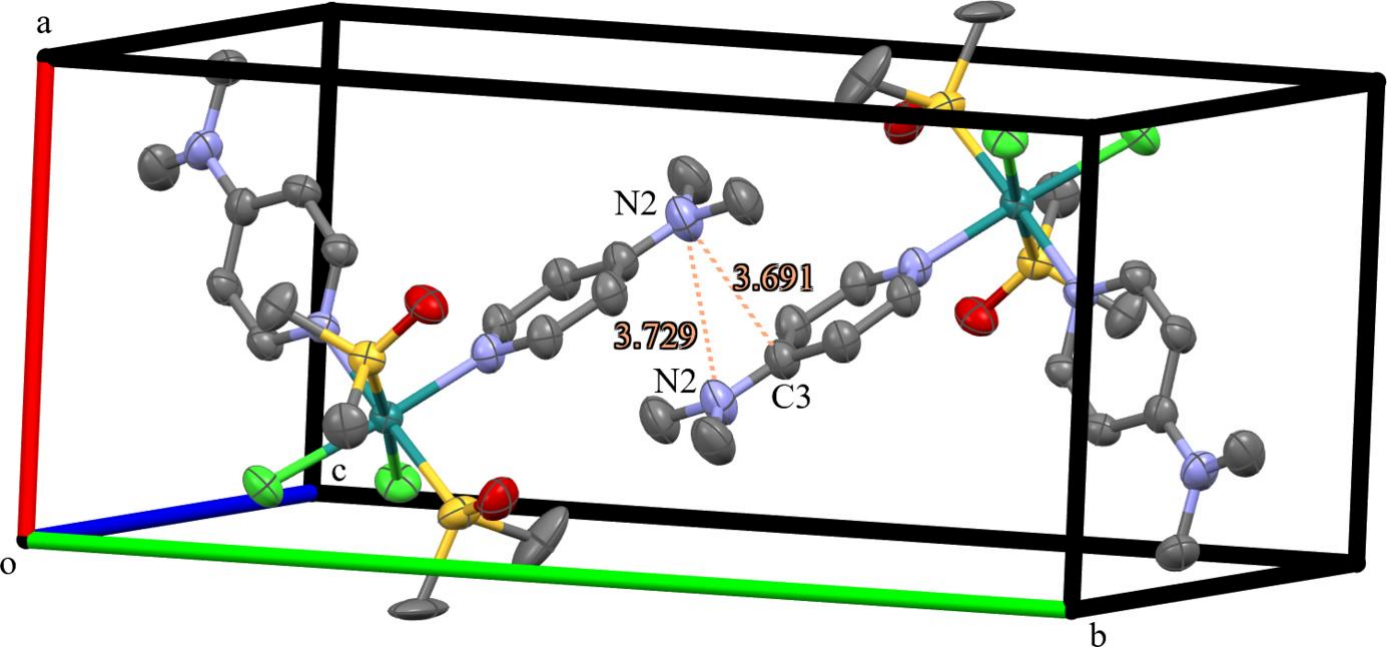
Displacement ellipsoid (50% probability level) representation of the packing of two mol­ecules of the title complex with inter­molecular distances given between the N2 atom and the C3 and N2 symmetry-related atoms (1 − *x*, 1 − *y*, 1 − *z*). Disorder and hydrogen atoms omitted for clarity.

The molecular complexes pack with one of the DMAP ligands offset above its symmetry-related counterpart, with inter­molecular distances of 3.691 (9) and 3.729 (8) Å, for N2⋯C3(1 − *x*, 1 − *y*, 1 − *z*) and N2⋯N2(1 − *x*, 1 − *y*, 1 − *z*), respectively (Fig. 2 ▸).

## Synthesis and crystallization

The formation of [Ru(DMAP)_6_]Cl_2_ was reported from the reaction of a fortyfold excess of DMAP with [Ru(dmso)_4_Cl_2_] in ethanol (Rossi *et al.*, 2008 ▸). With the aim toward the neutral tetra­kis­(DMAP) product, a general synthesis was followed, as described for *trans*-[RuCl_2_(pyrazine-κ*N*)_4_] (Carlucci *et al.*, 2002 ▸), where [RuCl_2_(dmso)_4_] and four equivalents of a pyridyl-based ligand are heated in toluene for multiple hours. This method has yielded *trans*-[RuCl_2_(NN)_4_] compounds with NN = 4-meth­oxy­pyridine (Reinheimer *et al.*, 2023 ▸) or pyrazine (Nesterov *et al.*, 2012 ▸). Rath and co-workers explored the effects of solvent polarity on the substitution of dmso and used a water/methanol solution of [Ru(dmso)_4_Cl_2_] and two equivalents of pyridine to form *cis,cis,cis*-[RuCl_2_(dmso-κ*S*)_2_(py)_2_] (Trivedi *et al.*, 2010 ▸). Unexpectedly, the title compound was synthesized using a non-polar solvent and four equivalents of a more basic pyridyl-type ligand, DMAP.

A Schlenk flask was charged with 0.1 g (0.2 mmol) of Ru(dmso)_4_Cl_2_ and 0.1 g (0.8 mmol) of DMAP, then combined with 20 ml of toluene. The flask was purged with N_2_ and the solution heated at reflux for 2 h. After slowly cooling, 0.11 g (90%) of a light-yellow solid was isolated after filtering in air and washing with ethanol. Brown prisms were grown by vapor diffusion between toluene and a chloro­form/toluene solution of the product.

## Refinement

Crystal data, data collection and structure refinement details are summarized in Table 1 ▸. The crystal was treated as a two-component twin with scales of 0.9262 (9) and 0.0738 (9). Two of the terminal methyl groups were modeled for disorder using restrained distances (SADI; same distances with standard deviation of 0.02 Å) for S1—C15 and S1—C16, with parts *A* and *B*, restraining these carbon atoms with roughly equivalent anisotropic displacement parameters (SIMU; similar *U*_ij_ components with standard deviation of 0.001 Å^2^), and summing the occupancies of each type of C to sum to one (FVAR and 1 - FVAR; Sheldrick, 2015*b* ▸).

## Supplementary Material

Crystal structure: contains datablock(s) I. DOI: 10.1107/S2414314624001913/bh4082sup1.cif

Structure factors: contains datablock(s) I. DOI: 10.1107/S2414314624001913/bh4082Isup2.hkl

CCDC reference: 2034089

Additional supporting information:  crystallographic information; 3D view; checkCIF report

## Figures and Tables

**Table 1:** Experimental details

Crystal data	
Chemical formula	[RuCl_2_(C_7_H_10_N_2_)_2_(C_2_H_6_OS)_2_]
*M*_r_	572.56
Crystal system, space group	Monoclinic, *P*2_1_/*n*
Temperature (K)	293
*a*, *b*, *c* (Å)	8.3125 (1), 18.9072 (4), 15.7906 (3)
β (°)	90.617 (2)
*V* (Å^3^)	2481.60 (8)
*Z*	4
Radiation type	Mo *K*α
μ (mm^−1^)	1.04
Crystal size (mm)	0.16 × 0.10 × 0.07

Data collection	
Diffractometer	XtaLAB Mini II
Absorption correction	Analytical [*CrysAlis PRO* (Rigaku OD, 2020 ▸) based on Clark & Reid (1995 ▸)]
*T*_min_, *T*_max_	0.969, 0.984
No. of measured, independent and observed [*I* > 2σ(*I*)] reflections	122167, 5695, 4795
*R*_int_	0.067
(sin θ/λ)_max_ (Å^−1^)	0.649

Refinement	
*R*[*F* ^2^ > 2σ(*F* ^2^)], *wR*(*F* ^2^), *S*	0.048, 0.148, 1.09
No. of reflections	5695
No. of parameters	293
No. of restraints	18
H-atom treatment	H-atom parameters constrained
Δρ_max_, Δρ_min_ (e Å^−3^)	1.35, −0.87

Computer programs: *CrysAlis PRO* (Rigaku OD, 2020 ▸), *SHELXT* (Sheldrick, 2015*a* ▸), *SHELXL* (Sheldrick, 2015*b* ▸), *OLEX2* (Dolomanov *et al.*, 2009 ▸), and *Mercury* (Macrae *et al.*, 2020 ▸).

## full crystallographic data

### Crystal data

**Table:**

[RuCl_2_(C_7_H_10_N_2_)_2_(C_2_H_6_OS)_2_]	*F*(000) = 1176
*M**_r_* = 572.56	*D*_x_ = 1.533 Mg m^−^^3^
Monoclinic, *P*2_1_/*n*	Mo *K*α radiation, λ = 0.71073 Å
*a* = 8.3125 (1) Å	Cell parameters from 31352 reflections
*b* = 18.9072 (4) Å	θ = 2.2–28.7°
*c* = 15.7906 (3) Å	µ = 1.04 mm^−^^1^
β = 90.617 (2)°	*T* = 293 K
*V* = 2481.60 (8) Å^3^	Block, brown
*Z* = 4	0.16 × 0.10 × 0.07 mm

### Data collection

**Table:**

XtaLAB Mini II diffractometer	4795 reflections with *I* > 2σ(*I*)
Radiation source: fine-focused sealed tube	*R*_int_ = 0.067
ω scans	θ_max_ = 27.5°, θ_min_ = 2.5°
Absorption correction: analytical [CrysAlisPro (Rigaku OD, 2020) based on Clark & Reid (1995)]	*h* = −10→10
*T*_min_ = 0.969, *T*_max_ = 0.984	*k* = −24→24
122167 measured reflections	*l* = −20→20
5695 independent reflections	

### Refinement

**Table:**

Refinement on *F*^2^	18 restraints
Least-squares matrix: full	Hydrogen site location: inferred from neighbouring sites
*R*[*F*^2^ > 2σ(*F*^2^)] = 0.048	H-atom parameters constrained
*wR*(*F*^2^) = 0.148	*w* = 1/[σ^2^(*F*_o_^2^) + (0.0631*P*)^2^ + 9.1602*P*] where *P* = (*F*_o_^2^ + 2*F*_c_^2^)/3
*S* = 1.09	(Δ/σ)_max_ = 0.001
5695 reflections	Δρ_max_ = 1.35 e Å^−^^3^
293 parameters	Δρ_min_ = −0.87 e Å^−^^3^

### Special details

**Table:**

Refinement. Refined as a 2-component twin.

### Fractional atomic coordinates and isotropic or equivalent isotropic displacement parameters (Å^2^)

**Table:**

	*x*	*y*	*z*	*U*_iso_*/*U*_eq_	Occ. (<1)
Ru1	0.73027 (5)	0.72633 (2)	0.75951 (3)	0.03186 (13)	
S2	0.59464 (17)	0.71392 (7)	0.88155 (8)	0.0373 (3)	
Cl2	0.88050 (19)	0.82299 (8)	0.82624 (10)	0.0511 (4)	
S1	0.91252 (18)	0.64259 (9)	0.79645 (11)	0.0499 (4)	
Cl1	0.8805 (2)	0.74749 (10)	0.63031 (10)	0.0531 (4)	
O1	0.8687 (6)	0.5892 (2)	0.8604 (3)	0.0576 (12)	
O2	0.4730 (5)	0.6575 (2)	0.8889 (3)	0.0524 (11)	
N3	0.5583 (5)	0.8030 (2)	0.7148 (3)	0.0339 (9)	
N2	0.3047 (7)	0.5048 (3)	0.5567 (4)	0.0571 (14)	
N4	0.2210 (7)	0.9497 (3)	0.6181 (4)	0.0508 (12)	
N1	0.5925 (6)	0.6498 (2)	0.6931 (3)	0.0404 (10)	
C9	0.2838 (7)	0.8364 (3)	0.6840 (3)	0.0404 (12)	
H9	0.175319	0.824777	0.687201	0.048*	
C8	0.3974 (7)	0.7900 (3)	0.7130 (4)	0.0381 (11)	
H8	0.362395	0.746412	0.732970	0.046*	
C3	0.3963 (8)	0.5519 (3)	0.6005 (4)	0.0453 (13)	
C10	0.3299 (7)	0.9020 (3)	0.6492 (3)	0.0405 (12)	
C5	0.5422 (7)	0.6618 (3)	0.6125 (3)	0.0410 (12)	
H5	0.575206	0.703469	0.586635	0.049*	
C12	0.6030 (7)	0.8656 (3)	0.6827 (4)	0.0434 (13)	
H12	0.712162	0.876319	0.682320	0.052*	
C11	0.4969 (7)	0.9152 (3)	0.6503 (4)	0.0461 (14)	
H11	0.536018	0.957625	0.628999	0.055*	
C14	0.2713 (10)	1.0068 (4)	0.5618 (5)	0.0648 (19)	
H14A	0.378701	1.021287	0.576635	0.097*	
H14B	0.199495	1.046257	0.567635	0.097*	
H14C	0.268760	0.990457	0.504287	0.097*	
C1	0.5460 (8)	0.5873 (3)	0.7265 (4)	0.0463 (13)	
H1	0.579416	0.576580	0.781458	0.056*	
C2	0.4523 (8)	0.5385 (3)	0.6841 (4)	0.0498 (14)	
H2	0.425382	0.496277	0.710649	0.060*	
C4	0.4461 (8)	0.6169 (3)	0.5664 (4)	0.0469 (14)	
H4	0.413423	0.629550	0.511941	0.056*	
C17	0.7239 (9)	0.7070 (4)	0.9729 (4)	0.0544 (16)	
H17A	0.783317	0.663560	0.970460	0.082*	
H17B	0.660093	0.707661	1.023245	0.082*	
H17C	0.797394	0.746193	0.973982	0.082*	
C7	0.2353 (11)	0.5231 (4)	0.4753 (5)	0.070 (2)	
H7A	0.132824	0.545369	0.483275	0.105*	
H7B	0.221327	0.480997	0.442004	0.105*	
H7C	0.305776	0.555062	0.446397	0.105*	
C13	0.0489 (8)	0.9328 (4)	0.6152 (5)	0.0585 (17)	
H13A	0.025195	0.905823	0.565108	0.088*	
H13B	−0.012379	0.975811	0.614316	0.088*	
H13C	0.021278	0.905643	0.664343	0.088*	
C6	0.2547 (10)	0.4391 (4)	0.5945 (5)	0.0665 (19)	
H6A	0.347232	0.414532	0.616460	0.100*	
H6B	0.202125	0.410330	0.552418	0.100*	
H6C	0.181547	0.448616	0.639694	0.100*	
C18	0.4956 (10)	0.7942 (4)	0.9103 (4)	0.0586 (18)	
H18A	0.568704	0.833165	0.904579	0.088*	
H18B	0.460866	0.791123	0.967999	0.088*	
H18C	0.403896	0.801449	0.873846	0.088*	
C16B	1.115 (3)	0.674 (3)	0.811 (4)	0.094 (9)	0.49 (7)
H16A	1.151237	0.695361	0.759029	0.141*	0.49 (7)
H16B	1.184112	0.635662	0.825903	0.141*	0.49 (7)
H16C	1.117362	0.709174	0.855091	0.141*	0.49 (7)
C15B	0.971 (3)	0.5886 (13)	0.7074 (8)	0.107 (7)	0.89 (5)
H15A	0.879157	0.562820	0.686573	0.161*	0.89 (5)
H15B	1.053119	0.555928	0.725013	0.161*	0.89 (5)
H15C	1.011517	0.618346	0.663205	0.161*	0.89 (5)
C16A	1.088 (4)	0.684 (2)	0.843 (3)	0.094 (9)	0.51 (7)
H16D	1.113528	0.726098	0.811056	0.141*	0.51 (7)
H16E	1.177123	0.652033	0.840928	0.141*	0.51 (7)
H16F	1.066494	0.696755	0.900281	0.141*	0.51 (7)
C15A	1.026 (19)	0.625 (9)	0.702 (5)	0.107 (7)	0.11 (5)
H15D	0.989372	0.655770	0.657266	0.161*	0.11 (5)
H15E	1.010593	0.576730	0.685221	0.161*	0.11 (5)
H15F	1.138045	0.633495	0.713066	0.161*	0.11 (5)

### Atomic displacement parameters (Å^2^)

**Table:**

	*U*^11^	*U*^22^	*U*^33^	*U*^12^	*U*^13^	*U*^23^
Ru1	0.0324 (2)	0.0340 (2)	0.02915 (19)	−0.00073 (16)	0.00050 (15)	0.00225 (15)
S2	0.0387 (7)	0.0418 (7)	0.0313 (6)	−0.0010 (5)	0.0018 (5)	0.0026 (5)
Cl2	0.0518 (8)	0.0528 (8)	0.0486 (8)	−0.0152 (7)	−0.0079 (6)	−0.0009 (6)
S1	0.0385 (7)	0.0533 (9)	0.0580 (9)	0.0114 (6)	0.0036 (7)	0.0098 (7)
Cl1	0.0510 (9)	0.0664 (9)	0.0421 (7)	−0.0077 (7)	0.0132 (6)	0.0031 (7)
O1	0.062 (3)	0.043 (2)	0.068 (3)	0.011 (2)	0.002 (2)	0.011 (2)
O2	0.047 (2)	0.061 (3)	0.050 (2)	−0.014 (2)	0.0099 (19)	0.001 (2)
N3	0.032 (2)	0.037 (2)	0.033 (2)	−0.0018 (18)	−0.0031 (17)	0.0026 (17)
N2	0.067 (4)	0.043 (3)	0.060 (3)	−0.015 (3)	−0.012 (3)	0.000 (2)
N4	0.054 (3)	0.041 (3)	0.057 (3)	0.008 (2)	−0.010 (3)	0.004 (2)
N1	0.049 (3)	0.036 (2)	0.037 (2)	−0.002 (2)	−0.003 (2)	0.0021 (19)
C9	0.035 (3)	0.047 (3)	0.039 (3)	−0.003 (2)	−0.003 (2)	0.002 (2)
C8	0.036 (3)	0.036 (3)	0.042 (3)	−0.005 (2)	0.000 (2)	0.007 (2)
C3	0.051 (3)	0.038 (3)	0.048 (3)	−0.005 (3)	−0.003 (3)	0.001 (2)
C10	0.048 (3)	0.037 (3)	0.036 (3)	0.002 (2)	−0.005 (2)	−0.001 (2)
C5	0.053 (3)	0.036 (3)	0.034 (3)	−0.006 (2)	−0.001 (2)	0.003 (2)
C12	0.040 (3)	0.037 (3)	0.053 (3)	−0.008 (2)	−0.003 (3)	0.007 (2)
C11	0.046 (3)	0.032 (3)	0.060 (4)	−0.007 (2)	−0.008 (3)	0.009 (2)
C14	0.073 (5)	0.045 (4)	0.076 (5)	0.002 (3)	−0.025 (4)	0.014 (3)
C1	0.061 (4)	0.038 (3)	0.040 (3)	−0.004 (3)	0.000 (3)	0.006 (2)
C2	0.064 (4)	0.037 (3)	0.048 (3)	−0.008 (3)	−0.002 (3)	0.007 (2)
C4	0.058 (4)	0.043 (3)	0.039 (3)	−0.003 (3)	−0.007 (3)	0.005 (2)
C17	0.068 (4)	0.063 (4)	0.032 (3)	0.001 (3)	−0.010 (3)	0.008 (3)
C7	0.075 (5)	0.065 (5)	0.070 (5)	−0.015 (4)	−0.025 (4)	−0.001 (4)
C13	0.046 (3)	0.059 (4)	0.071 (4)	0.012 (3)	−0.012 (3)	−0.002 (3)
C6	0.072 (5)	0.049 (4)	0.078 (5)	−0.019 (4)	−0.009 (4)	0.000 (3)
C18	0.078 (5)	0.054 (4)	0.044 (3)	0.021 (3)	0.012 (3)	0.000 (3)
C16B	0.014 (6)	0.081 (12)	0.19 (3)	0.005 (9)	0.009 (10)	0.041 (16)
C15B	0.156 (13)	0.095 (13)	0.072 (6)	0.079 (11)	0.040 (7)	0.004 (7)
C16A	0.014 (6)	0.081 (12)	0.19 (3)	0.005 (9)	0.009 (10)	0.041 (16)
C15A	0.156 (13)	0.095 (13)	0.072 (6)	0.079 (12)	0.040 (7)	0.004 (7)

### Geometric parameters (Å, º)

**Table:**

Ru1—S2	2.2553 (14)	C11—H11	0.9300
Ru1—Cl2	2.4456 (15)	C14—H14A	0.9600
Ru1—S1	2.2635 (15)	C14—H14B	0.9600
Ru1—Cl1	2.4366 (15)	C14—H14C	0.9600
Ru1—N3	2.150 (4)	C1—H1	0.9300
Ru1—N1	2.117 (5)	C1—C2	1.376 (9)
S2—O2	1.476 (4)	C2—H2	0.9300
S2—C17	1.794 (6)	C4—H4	0.9300
S2—C18	1.788 (6)	C17—H17A	0.9600
S1—O1	1.476 (5)	C17—H17B	0.9600
S1—C16B	1.799 (13)	C17—H17C	0.9600
S1—C15B	1.809 (9)	C7—H7A	0.9600
S1—C16A	1.804 (14)	C7—H7B	0.9600
S1—C15A	1.804 (18)	C7—H7C	0.9600
N3—C8	1.360 (7)	C13—H13A	0.9600
N3—C12	1.341 (7)	C13—H13B	0.9600
N2—C3	1.355 (8)	C13—H13C	0.9600
N2—C7	1.446 (9)	C6—H6A	0.9600
N2—C6	1.442 (9)	C6—H6B	0.9600
N4—C10	1.364 (7)	C6—H6C	0.9600
N4—C14	1.463 (9)	C18—H18A	0.9600
N4—C13	1.466 (8)	C18—H18B	0.9600
N1—C5	1.355 (7)	C18—H18C	0.9600
N1—C1	1.352 (7)	C16B—H16A	0.9600
C9—H9	0.9300	C16B—H16B	0.9600
C9—C8	1.366 (8)	C16B—H16C	0.9600
C9—C10	1.412 (8)	C15B—H15A	0.9600
C8—H8	0.9300	C15B—H15B	0.9600
C3—C2	1.419 (9)	C15B—H15C	0.9600
C3—C4	1.406 (8)	C16A—H16D	0.9600
C10—C11	1.410 (8)	C16A—H16E	0.9600
C5—H5	0.9300	C16A—H16F	0.9600
C5—C4	1.370 (8)	C15A—H15D	0.9600
C12—H12	0.9300	C15A—H15E	0.9600
C12—C11	1.381 (8)	C15A—H15F	0.9600
			
S2—Ru1—Cl2	88.12 (5)	N4—C14—H14C	109.5
S2—Ru1—S1	92.66 (6)	H14A—C14—H14B	109.5
S2—Ru1—Cl1	176.35 (6)	H14A—C14—H14C	109.5
S1—Ru1—Cl2	94.27 (6)	H14B—C14—H14C	109.5
S1—Ru1—Cl1	89.02 (6)	N1—C1—H1	118.0
Cl1—Ru1—Cl2	88.52 (6)	N1—C1—C2	123.9 (6)
N3—Ru1—S2	90.81 (12)	C2—C1—H1	118.0
N3—Ru1—Cl2	88.40 (12)	C3—C2—H2	119.6
N3—Ru1—S1	175.69 (12)	C1—C2—C3	120.8 (5)
N3—Ru1—Cl1	87.66 (12)	C1—C2—H2	119.6
N1—Ru1—S2	94.54 (13)	C3—C4—H4	119.6
N1—Ru1—Cl2	174.55 (13)	C5—C4—C3	120.7 (5)
N1—Ru1—S1	90.36 (14)	C5—C4—H4	119.6
N1—Ru1—Cl1	88.68 (14)	S2—C17—H17A	109.5
N1—Ru1—N3	86.82 (18)	S2—C17—H17B	109.5
O2—S2—Ru1	119.47 (19)	S2—C17—H17C	109.5
O2—S2—C17	106.8 (3)	H17A—C17—H17B	109.5
O2—S2—C18	106.0 (3)	H17A—C17—H17C	109.5
C17—S2—Ru1	113.2 (2)	H17B—C17—H17C	109.5
C18—S2—Ru1	111.4 (2)	N2—C7—H7A	109.5
C18—S2—C17	97.5 (3)	N2—C7—H7B	109.5
O1—S1—Ru1	119.0 (2)	N2—C7—H7C	109.5
O1—S1—C16B	112 (2)	H7A—C7—H7B	109.5
O1—S1—C15B	102.5 (9)	H7A—C7—H7C	109.5
O1—S1—C16A	103.1 (19)	H7B—C7—H7C	109.5
O1—S1—C15A	125 (5)	N4—C13—H13A	109.5
C16B—S1—Ru1	114.9 (18)	N4—C13—H13B	109.5
C16B—S1—C15B	92 (2)	N4—C13—H13C	109.5
C15B—S1—Ru1	112.3 (5)	H13A—C13—H13B	109.5
C16A—S1—Ru1	109.5 (16)	H13A—C13—H13C	109.5
C15A—S1—Ru1	106 (4)	H13B—C13—H13C	109.5
C15A—S1—C16A	89 (7)	N2—C6—H6A	109.5
C8—N3—Ru1	122.3 (4)	N2—C6—H6B	109.5
C12—N3—Ru1	122.2 (4)	N2—C6—H6C	109.5
C12—N3—C8	115.4 (5)	H6A—C6—H6B	109.5
C3—N2—C7	120.9 (6)	H6A—C6—H6C	109.5
C3—N2—C6	121.2 (6)	H6B—C6—H6C	109.5
C6—N2—C7	117.4 (6)	S2—C18—H18A	109.5
C10—N4—C14	120.9 (6)	S2—C18—H18B	109.5
C10—N4—C13	120.7 (5)	S2—C18—H18C	109.5
C14—N4—C13	115.3 (5)	H18A—C18—H18B	109.5
C5—N1—Ru1	120.7 (4)	H18A—C18—H18C	109.5
C1—N1—Ru1	124.0 (4)	H18B—C18—H18C	109.5
C1—N1—C5	115.3 (5)	S1—C16B—H16A	109.5
C8—C9—H9	119.8	S1—C16B—H16B	109.5
C8—C9—C10	120.4 (5)	S1—C16B—H16C	109.5
C10—C9—H9	119.8	H16A—C16B—H16B	109.5
N3—C8—C9	124.5 (5)	H16A—C16B—H16C	109.5
N3—C8—H8	117.7	H16B—C16B—H16C	109.5
C9—C8—H8	117.7	S1—C15B—H15A	109.5
N2—C3—C2	122.3 (6)	S1—C15B—H15B	109.5
N2—C3—C4	123.0 (6)	S1—C15B—H15C	109.5
C4—C3—C2	114.7 (5)	H15A—C15B—H15B	109.5
N4—C10—C9	122.6 (5)	H15A—C15B—H15C	109.5
N4—C10—C11	122.5 (5)	H15B—C15B—H15C	109.5
C11—C10—C9	114.9 (5)	S1—C16A—H16D	109.5
N1—C5—H5	117.7	S1—C16A—H16E	109.5
N1—C5—C4	124.6 (5)	S1—C16A—H16F	109.5
C4—C5—H5	117.7	H16D—C16A—H16E	109.5
N3—C12—H12	117.9	H16D—C16A—H16F	109.5
N3—C12—C11	124.1 (5)	H16E—C16A—H16F	109.5
C11—C12—H12	117.9	S1—C15A—H15D	109.5
C10—C11—H11	119.7	S1—C15A—H15E	109.5
C12—C11—C10	120.6 (5)	S1—C15A—H15F	109.5
C12—C11—H11	119.7	H15D—C15A—H15E	109.5
N4—C14—H14A	109.5	H15D—C15A—H15F	109.5
N4—C14—H14B	109.5	H15E—C15A—H15F	109.5
			
Ru1—N3—C8—C9	−178.9 (4)	C10—C9—C8—N3	3.1 (9)
Ru1—N3—C12—C11	177.3 (5)	C5—N1—C1—C2	−1.0 (9)
Ru1—N1—C5—C4	−176.6 (5)	C12—N3—C8—C9	−2.1 (8)
Ru1—N1—C1—C2	177.9 (5)	C14—N4—C10—C9	−162.6 (6)
N3—C12—C11—C10	0.0 (10)	C14—N4—C10—C11	18.7 (9)
N2—C3—C2—C1	179.6 (6)	C1—N1—C5—C4	2.4 (9)
N2—C3—C4—C5	−178.3 (6)	C2—C3—C4—C5	0.5 (9)
N4—C10—C11—C12	179.6 (6)	C4—C3—C2—C1	0.8 (10)
N1—C5—C4—C3	−2.2 (10)	C7—N2—C3—C2	173.3 (7)
N1—C1—C2—C3	−0.6 (10)	C7—N2—C3—C4	−8.0 (11)
C9—C10—C11—C12	0.8 (9)	C13—N4—C10—C9	−3.5 (9)
C8—N3—C12—C11	0.6 (9)	C13—N4—C10—C11	177.7 (6)
C8—C9—C10—N4	178.9 (6)	C6—N2—C3—C2	2.0 (11)
C8—C9—C10—C11	−2.2 (8)	C6—N2—C3—C4	−179.3 (7)
